# Natural selection fluctuates at an extremely fine spatial scale inside a wild population of snapdragon plants

**DOI:** 10.1111/evo.14359

**Published:** 2021-10-01

**Authors:** Pascal Marrot, Mathieu Latutrie, Jésaëlle Piquet, Benoit Pujol

**Affiliations:** ^1^ PSL Université Paris: EPHE‐UPVD‐CNRS, USR 3278 CRIOBE Université de Perpignan Perpignan 66860 France

**Keywords:** *Antirrhinum majus* L., environmental heterogeneity, fitness function, natural selection, snapdragon, spatial variation

## Abstract

Spatial variation in natural selection is expected to shape phenotypic variation of wild populations and drive their evolution. Although evidence of phenotypic divergence across populations experiencing different selection regimes is abundant, investigations of intrapopulation variation in selection pressures remain rare. Fine‐grained spatial environmental heterogeneity can be expected to influence selective forces within a wild population and thereby alter its fitness function by producing multiple fitness optima at a fine spatial scale. Here, we tested this hypothesis in a wild population of snapdragon plants living on an extremely small island in southern France (about 7500 m^2^). We estimated the spline‐based fitness function linking individuals’ fitness and five morphological traits in interaction with three spatially variable ecological drivers. We found that selection acting on several traits varied both in magnitude and direction in response to environmental variables at the scale of a meter. Our findings illustrate how different phenotypes can be selected at different locations within a population in response to environmental variation. Investigating spatial variation in selection within a population, in association with ecological conditions, represents an opportunity to identify putative ecological drivers of selection in the wild.

Evidence for natural selection having shaped phenotypic variation in natural populations can be found in many species (Endler 1986). Several empirical studies showed that natural selection fluctuates in time and space in many taxa, for example, plants (González et al. 2019), mammals (Coltman et al. 1999), birds (Grant and Grant 2002; Marrot et al. 2018), and arthropods (O'Hara 2005). Spatiotemporal fluctuations of selection pressures have the potential to maintain phenotypic variation between populations (Felsenstein 1976; Bell 2010). Spatial variation in selection coefficients has been evaluated between wild populations experiencing different environmental conditions where different selection regimes can drive the phenotypic and genetic divergence of populations and thereby maintain heritable interpopulation phenotypic variation (Siepielski et al. 2013). This theoretical framework is well suited to explain the presence of heritable phenotypic variation across populations. However, spatial variation in selection coefficients between populations cannot explain heritable phenotypic variation in the presence of selection acting at the intrapopulation scale. The magnitude and direction of selection coefficients are generally assumed to be homogenous within a population. Many studies showed that genetic variation for fitness‐related traits can be found at a fine spatial scale within wild populations (Postma and Van Noordwijk 2005; Garroway et al. 2013). Yet spatial variation in selection is rarely tested at the intrapopulation scale, although it could explain genetic variation in fitness within wild populations (Garant et al. 2007; Quinn et al. 2009; Baythavong 2011; Mojica et al. 2012; Bouwhuis et al. 2015).

Exploring intrapopulation variation in selection strength represents a great opportunity to understand the ecological causes of natural selection in the wild (Wade and Kalisz 1990). Several authors pointed out the need to go beyond the estimation of natural selection acting on traits by identifying the ecological agents that drive the fitness‐trait relationship (MacColl 2011; Caruso et al. 2017). To cite MacColl (2011): “*natural selection is not the cause of evolution, it is a process*.” This process is driven by ecological variables that often remain unidentified. During the last three decades, ecological agents of selection have been identified in several taxa (Caruso et al. 2017) of plants (Lau 2008; Caruso et al. 2019), fishes (Barrett 2010), and birds (Grant and Grant 2006). For example, Benkman (2003) identified the link between selection acting on bill depth in wild populations of different red crossbill species (*Loxia curvirostra*) and the conifer species they feed on. Although experimental approaches are useful to identify agents of selection (Wade and Kalisz 1990), most selection gradients measured in the wild remain unrelated to their ecological drivers. This is possibly the consequence of the high sampling effort required to build long‐term or spatially variable datasets that are necessary to track selection changes over time or space in wild populations. Also, investigating ecological drivers of fine‐scale spatial variation in selection requires identifying and sampling potential ecological variables that fluctuate within wild populations, which remains rare in previous studies.

Traditionally, the relationship between selection and its ecological drivers has been explored by quantifying the sensitivity of selection coefficients (differentials or gradients) to ecological variables (Chevin et al. 2010; Gamelon et al. 2018). This method requires including an interaction term between the focal trait and a potential environmental variable in the classical Lande and Arnold (1983) linear regression. However, this approach has two major issues. First, it assumes that the fitness‐trait relationship (the fitness function) and environmental variables follow a linear (or quadratic) shape, leading to numerous statistical problems when the form of the fitness function does not follow this assumption (Mitchell‐Olds and Shaw 1987; Schluter 1988). An alternative to this approach has been proposed by Schluter (1988), based on nonparametric spline‐based approximation of the fitness function. Although spline‐based fitness functions are useful to explore the shape of the fitness‐trait relationship, this approach has not been used often by ecologists because standardized selection gradients cannot be extracted from these functions (but see Morrissey and Sakrejda [2013] for a method allowing to extract selection gradients from nonlinear fitness function). Second, a significant effect of the environment on the selection coefficient cannot be interpreted as the signature of varying selection driven by a specific ecological variable (Hunter et al. 2018). This is because selection coefficients represent the slope of the fitness function (Lande and Arnold 1983; Arnold 2003) that links the standardized phenotype (zero mean and unit variance) of individuals to their relative fitness (i.e., standardization by the mean fitness). The standardization of the phenotype and fitness of individuals does not allow interpreting significant variation in selection coefficients as a fluctuation in the slope between the population mean fitness and phenotype. In fact, such variation could be the signature of variation in mean fitness, mean phenotype, or phenotypic variance (Chevin and Haller 2014; Gamelon et al. 2018; Hunter et al. 2018). This problem is inherent to the standardization of the phenotype (by the variance or the mean) and relative fitness (by the mean or the maximum) when calculating selection coefficients using the classical Lande and Arnold (1983) approach.

Here, we constructed the environmentally explicit spline‐based fitness function of a wild population of snapdragon plants (*Antirrhinum majus* L.) that lives on a small island in southern France. Their habitat exhibits a strong variation in terms of both biotic (vegetation cover, local density in conspecifics) and abiotic (substrate type) ecological variables despite a relatively small spatial scale (about 7500 m^2^). This represented an opportunity to test for the impact of ecological variation on natural selection acting on five morphological traits within a wild plant population. Contrary to the classical Lande and Arnold quadratic regression, our fitness function linked the absolute fitness of individuals to their unstandardized traits values and environmental variation, allowing to directly visualize the nonlinear impact of each environmental variable on selection.

## Material and Methods

### STUDY SYSTEM AND SITE

*Antirrhinum majus* L. (Plantaginaceae) is a hermaphroditic, self‐incompatible, short‐lived perennial (only 28% of plants survived more than one year), which produces annual inflorescences with zygomorphic flowers (pollinated by bumblebees). Two interfertile subspecies of *A. majus* occur in southern France; *A. m. pseudomajus* and *A. m. striatum* that harbor magenta and yellow flowers, respectively (Andalo et al. 2010). Although geographically separated (Khimoun et al. 2011), they inhabit a similar range of environmental conditions that consists of limestone or siliceous habitats with contrasted moisture regimes where they form restricted patches that thrive in rocky outcrops and screes, meadows, and disturbed habitats such as roadside and railway embankments (Khimoun et al. 2013). Here, we focus on a particular *A. m. pseudomajus* population monitored over multiple years on a small island (about 7500 m^2^) in the Bages lagoon (Southern France, 43°05ʹ30ʺ N, 3°00ʹ07ʺ E) that is characterized by strong ecological heterogeneity. In this population, snapdragon plants can be found on amid grass, screes, and on rocks that occur in multiple places across the island. In 2015, 2016, and 2017, all the reproductively mature snapdragon plants growing in this population have been monitored. The geographic coordinates of each individual were recorded with high precision centimeter‐level GNSS material (Trimble Geo7X). We also measured several phenotypic traits on each plant: the total number of leaves, the total number of branches, the number of stems, the mean internode distance between two subsequent nodes (averaged among all stems, in mm), and the height (height of the longest stem, in cm). We chose these traits because of their potential influence on fitness given the fact that snapdragon flowers are produced on the upper part of the stem. Snapdragon flowers are terminal flowers that prevent further growth of vegetative organs on the stem on which they grew. Further vegetative growth and additional inflorescences are restricted to axillary parts of the main stem such as branches or other stems growing from the basis. Vegetative growth might influence negatively the production of flowers through trade‐offs of biomass allocation to vegetative versus reproductive growth but is more likely to be positively correlated to fitness if bigger plants produce more flowers that turn into fruits. The total number of fruits produced by each plant was recorded and considered as the proxy of fitness in the following analyses of selection. Every proxy of fitness suffers limitations and the number of fruits is no exception because it does not take into account the successful germination of progeny and strongly reflects the female reproductive success, ignoring the male reproductive success.

### ENVIRONMENTAL VARIABLES

We characterized the specific environment of each individual by recording three environmental variables: the substrate type, the vegetation coverage, and the density in conspecifics. The substrate type corresponds to the substrate that the plant is rooted in, which can be interpreted as a proxy for below ground space and resources available for each individual (Supporting Information 1). This abiotic microenvironment has been evaluated on site by using a gradient of soil composition ranging from 1 to 4 (1 = bedrock, 2 = large rocks, 3 = rocky soil, 4 = soil), which allowed us to consider soil content in the substrate as a continuous variable. Our choice of considering this variable as continuous was motivated by the natural design of the study site. Indeed, the distribution of the different substrate types was not randomized along the gradient of vegetation coverage (e.g., a high vegetation coverage was rarely associated with a bed rock substrate type), which prevented obtaining reliable results when using categorical variables. The closest most statistically appropriate reliable approach was to use a continuous variable. We acknowledge the limited availability of environmental combinations in this wild plant population. Caution should be taken when interpreting the results because there is no a priori biological reason, for example, to consider that the difference between “Bed Rock” and “Large Rocks” is equal to the difference between “Rocky Soil” and “Soil.” The vegetation coverage (dominated by grass and shrubs) corresponded to the coverage in foliage within a 150‐mm radius around each individual (Supporting Information 1). This measurement is expressed in percentage (approximated to the nearest 10%) and was assessed on the basis of photos taken in the field from above the top of each individual. The center of the 150‐mm‐radius circle corresponded to the point on the ground where the plant is rooting. The circle was drawn using Image J software on the basis of a standardized 75‐mm‐wide post‐it placed on the ground close to the individual or a real plastic circle directly put on the ground around the plant in the field whenever possible. The percentage of vegetation coverage was visually assessed within the circle. This environmental variable reflects partly the heterospecific local density and biomass, a type of biotic microenvironment that might affect the growth of plants through resource competition (Reader et al. 1994). The density of conspecifics corresponded to the number of snapdragon plants within a 1‐m radius around each individual. This measurement was estimated on the basis of their geographic coordinates after constructing the Euclidean distance matrix among all individuals of the population. This biotic environmental variable might reflect local snapdragon plant interactions in terms of resource limitation and facilitation or competition for pollinators that have been shown to affect their reproductive success (Inaba et al. 2010; Tastard et al. 2012).

### STATISTICAL ANALYSES

Statistical analyses were conducted on a dataset including 2383 plants (see statistical distribution of all variables used in Supporting Information 2) recorded only one year and first‐year measurements of plants recorded multiple years (i.e., our dataset contained only one record by individual). We constructed the nonlinear fitness function relating individual absolute fitness to traits in interaction with environmental variables. Following the method outlined in Morrissey and Sakrejda 2013, we used a spline‐based fitness function (i.e., Generalized Additive Models [GAM], Wood 2017) that allows the flexible inference of the shape of the function linking fitness and phenotypes (Schluter 1988). Here, we estimated the fitness function relating individual fitness (the number of fruits) to other variables as a smoother‐based function of the number of leaves, branches, stems, the internode distance, height, substrate type, vegetation coverage, and density in conspecifics. Although the effect of traits on fitness translates the selection acting on traits, the inclusion of our three environmental variables in the fitness function allowed us to control for their direct effect on fitness. We added the full smoother‐based tensor product of each trait with each environmental variable in the model, for a total of 15 full tensor products. The monitoring year was added as a covariable to take into account the significant annual fluctuation in fitness (*P* < 0.001, estimated with a generalized linear model). Because fitness was significantly spatially autocorrelated in our population (Moran test *P* < 0.001), we included a smooth‐based tensor product of the longitude and latitude to control for spatial autocorrelation (Supporting information 3). This model was fitted using a Poisson distribution.

We estimated the linear and quadratic standardized selection gradients of each trait by obtaining the first and second (partial) derivatives of the fitness function using the package GSG in R (Morrissey and Sakrejda 2013). Using the same approach, we estimated the gradient related to the direct effect of the three environmental variables on fitness (called hereafter the “environmental gradient”). Finally, we tested the effect of environmental variables on selection by estimating the selection gradients associated with each of the 15 full tensor products between our three environmental variables and our five traits (hereafter named “ecological selection gradients”). The standard errors associated with each selection gradient were obtained by using parametric bootstrap (1000 bootstraps) and *P*‐values were calculated based on the proportion of estimates above and below an a priori null value (see Morrissey and Sakrejda [2013] for further details).

## Results

The fitness function that we constructed explained 71.3% of the variation of the absolute fitness of individuals in the wild population of snapdragon plants. The standardized environmental gradients of substrate type, vegetation coverage, and conspecifics density corresponding to the linear effect of the environmental variables on fitness were equal to −0.228 ± 0.018 (*P* < 0.001), −0.036 ± 0.028 (*P* = 0.142), and −0.151 ± 0.031 (*P* < 0.001), respectively, traducing a significant lower fitness of plants living in a soil substrate type and at a high density of conspecifics. We detected positive directional selection (via standardized linear selection gradients; Table 1) for the number of branches (0.057 ± 0.020), the number of stems (0.231 ± 0.016), and height (0.727 ± 0.011), and negative directional selection for the number of leaves (−0.092 ± 0.021) and the internode distance (−0.151 ± 0.016). Nonlinear selection (via quadratic standardized selection gradients) was detected for all the traits, except for the internode distance (Table 1). Regarding directional and quadratic selection gradients, taller plants with more leaves, branches, stems, and shorter internode distances produced more fruits (Supporting Information 4).

**Table 1:** Linear, quadratic, and ecological standardized selection gradients (± SE) of number of leaves, number of branches, number of stems, internode distance, and height. *β* and *γ* represent the linear and quadratic selection gradients. *γ*_z.substrate type_, *γ*_z.vegetation coverage_, and *γ*_z.local density_ represent the ecological selection gradients between each trait and substrate type (considered as a continuous variable, from 1 = bed rock to 4 = soil), vegetation coverage, and local density, respectively. In bold, the significant (*P* < 0.001) selection gradients

	Number of leaves	Number of branches	Number of stems	Internodes distance	Height
*β*	**−0.092 (± 0.021)**	**0.057 (± 0.020)**	**0.231 (± 0.016)**	**−0.151 (± 0.016)**	**0.727 (± 0.011)**
*γ*	**0.221 (± 0.041)**	**0.267 (± 0.059)**	**0.061 (± 0.017)**	−0.012 (± 0.040)	**0.281 (± 0.020)**
*γ*_z.substrate type_	**−0.102 (± 0.028)**	**0.106 (± 0.029)**	−0.028 (± 0.022)	**0.081 (± 0.022)**	**−0.161 (± 0.020)**
*γ*_z.vegetation coverage_	0.000 (± 0.025)	0.037 (± 0.025)	0.010 (± 0.023)	−0.021 (± 0.027)	**−0.068 (± 0.024)**
*γ*_z.local density_	−0.076 (± 0.042)	0.060 (± 0.041)	0.004 (± 0.031)	−0.032 (± 0.026)	**−0.080 (± 0.032)**

**Figure 1:**
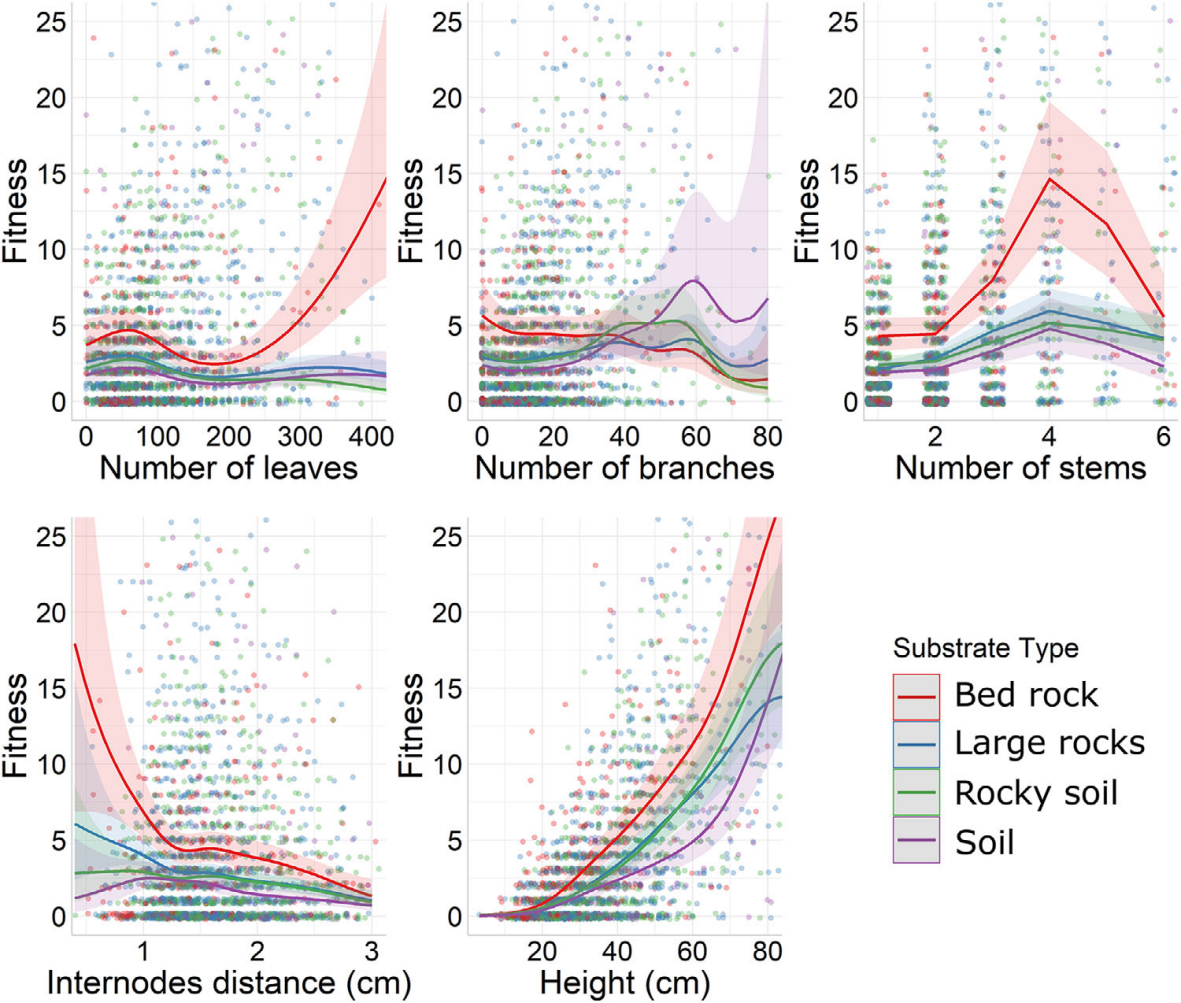
Predicted number of fruits (fitness and its 95% confidence intervals) extracted from the fitness function, depending on a focal trait (number of leaves, number of branches, number of stems, internodes distance, and height) in our four different substrate types, holding all the nonfocal variables constant with a value of number of leaves = 82, number of branches = 14, number of stems = 2, internode distance = 1.61 cm, height = 37.34, vegetation coverage = 50%, conspecifics density = 2, year = 2016, latitude = 43.11, and longitude = 2.99. These values are corresponding to the mean value of each variable in the population. The points represent the raw data.

**Figure 2:**
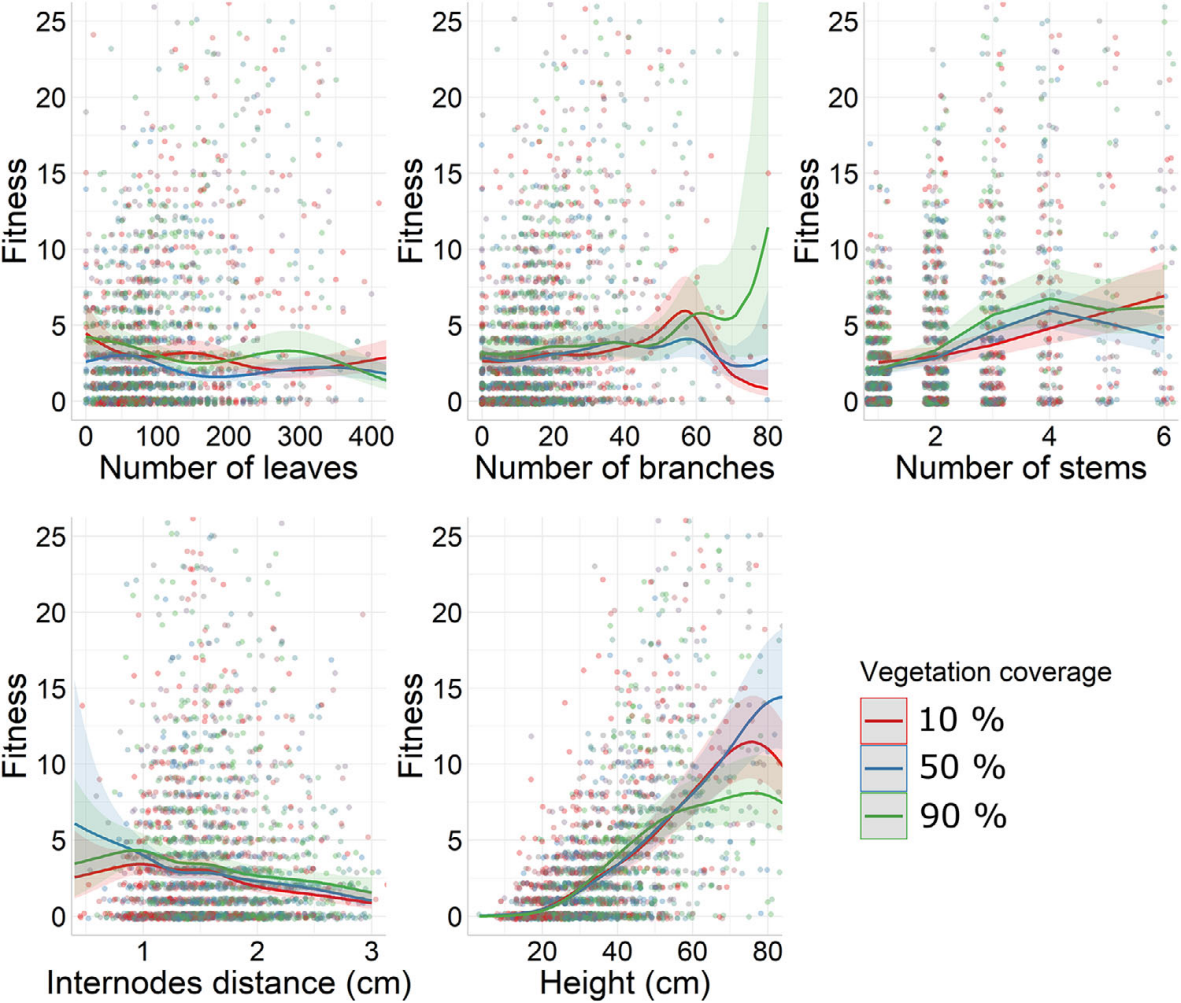
Predicted number of fruits (fitness and its 95% confidence intervals) extracted from the fitness function, depending on a focal trait (number of leaves, number of branches, number of stems, internodes distance, and height) in three different values of vegetation coverage (10%, 50%, and 90%), holding all the nonfocal variables constant with a value of number of leaves = 82, number of branches = 14, number of stems = 2, internode distance = 1.61 cm, height = 37.34, conspecifics density = 2, year = 2016, latitude = 43.11, longitude = 2.99, and a rocky soil substrate type. These values are corresponding to the mean value of each variable in the population. The points represent the raw data.

**Figure 3:**
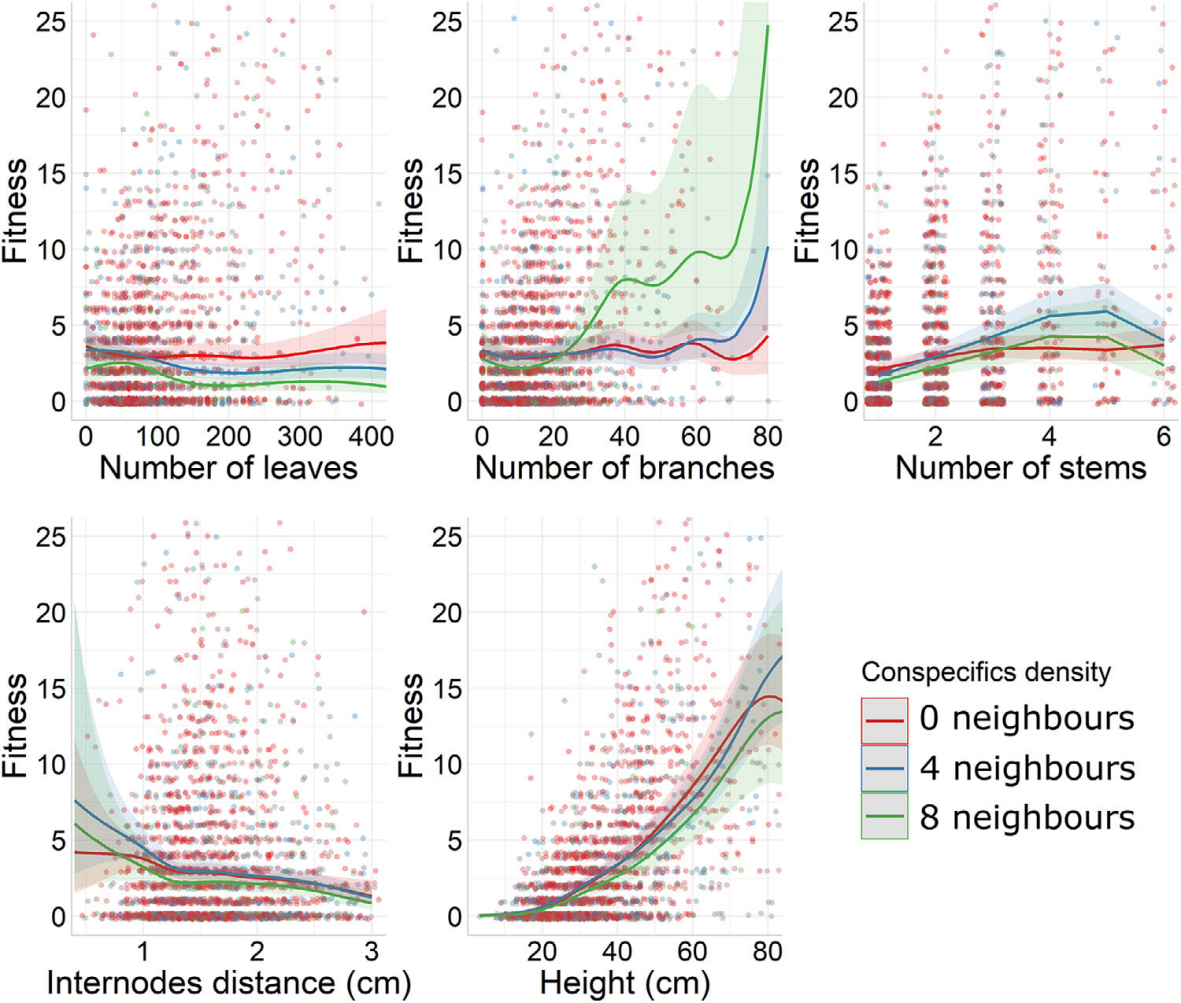
Predicted number of fruits (fitness and its 95% confidence intervals) extracted from the fitness function, depending on a focal trait (number of leaves, number of branches, number of stems, internodes distance, and height) in three different values of conspecifics density (0, 4, and 8 neighbors), holding all the nonfocal variables constant with a value of number of leaves = 82, number of branches = 14, number of stems = 2, internode distance = 1.61 cm, height = 37.34, vegetation coverage = 50%, year = 2016, latitude = 43.11, longitude = 2.99, and a rocky soil substrate type. These values are corresponding to the mean value of each variable in the population. The points represent the raw data.

The relationship between selection and environmental variables has been tested using the ecological selection gradients and illustrated in Figures 1, 2, 3 for a few values of environmental variation. All the standardized ecological selection gradients between a given trait and the substrate type were significant, excluding the number of stems (Table 1). Although these gradients were negative for the number of leaves and the height, they were positive for the number of branches and the internode distance (Table 1). This means that increased soil content in the substrate type was associated with weaker selection acting on the number of leaves, the internode distance, height, and stronger selection acting on the number of branches (Fig. 1). Most substantial changes in selection were detected for the number of leaves that was associated with a directional positive selection only on the bed rock substrate type (Fig. 1). Caution must be taken when interpreting the ecological selection gradients between the traits and the substrate type as they represent a linear interaction between the soil content, the traits, and fitness because we considered the substrate type as a continuous variable. Therefore, we were not able to detect a nonlinear effect of substrate type on selection, for example, a selective effect of living in a rocky soil substrate type compared to the three other substrate types.

The standardized ecological selection gradients between a given trait and the two other environmental variables (vegetation coverage [Fig. 2] and conspecifics density [Fig. 3]) were significant and negative only for height (Table 1). This means that a small number of neighbors with a 1‐m radius and a low value of vegetation coverage were associated with stronger selection acting on height (Figs. 2 and 3).

## Discussion

Our results showed that the snapdragon plant population in southern France used in this study was under selection pressures acting on the number of leaves, the number of branches, the number of stems, the internode distance, and height. This is not surprising because snapdragon plants are characterized by terminal flowering. This result was expected because bigger stems are more likely to produce more flowers leading to potentially more fruits (personal observation) even if the production of a high number of fruits by plants producing a high number of flowers might be pollen limited (Andalo et al. 2010). Plants harboring more stems (when considering stems with the same height) can also produce more flowers.

Our calculation of the spline‐based fitness function relating the absolute fitness of individuals to their phenotypic trait values in interaction with our three environmental variables highlighted the complex relationship between environmental variation and selection in our population. The first relevant result was that the relationship between fitness and traits varied inside our population despite its relatively small spatial scale. This represents rare evidence of spatial variation of natural selection within a population of plants pollinated by insects at the scale of a few meters (but see Garant et al. [2007], Quinn et al. [2009], Baythavong [2011], Mojica et al. [2012], and Bouwhuis et al. [2015] for examples of spatial variation in the fitness function within relatively small wild populations). This finding has two main implications.

First, it implies that selection gradients estimated at the population scale reflect only partly the scope of ecological conditions that shape selection in the population. For example, considering the linear (*β* = −0.092 ± 0.021) and quadratic (*γ* = 0.221 ± 0.041) selection gradient acting on the number of leaves and the visualization of the fitness‐trait relationship at the population scale (Supporting Information 4), an analysis of selection at the population scale would necessarily conclude that having a high number of leaves is not an advantage in this species. Although this interpretation is true on *average* at the population scale, our fitness function shows that the selective advantage having a high number of leaves is only true in the bed rock substrate type the plants are rooting in (Fig. 1). This finding does not alter a potential prediction of the evolutionary trajectory of the population under selection because traditional estimates of selection at the population scale implicitly integrate these microvariations of the environment. However, our environmental‐explicit fitness function allows to reach a better understanding of the ecological context that promotes selection in this species.

Second, such fine‐scale variation in selection regime means that different phenotypes are advantaged by selection at different locations, which could ultimately promote microgeographic adaptation (Richardson et al. 2014). In order to assess spatially autocorrelated natural selection, which was identified as a major driver of microgeographic adaptation by Richardson et al. (2014), it is necessary to consider the dispersal capacity of the species. In our population, the mean distance between the two parents of each individual is 14.5 ± 17.5 m (with 5% of parents distanced from each other by more than 50 m), which is enough to maintain constant gene flow between different patches of environment (distanced from each other by less than 10–15 m, pers. obs.) and prevent any microgeographic adaptation in response to the fine‐scale spatial variation of selection.

Beyond spatial variation of selection, our approach identified associated ecological drivers (see also the sensitivity analysis proposed by Hunter et al. [2018] for another powerful approach allowing to explore the drivers of selection). Among the three ecological variables that we analyzed, the shape of the fitness function was mostly altered by the substrate type. Specifically, increased soil content in the substrate type was associated with weaker selection acting on number of leaves, internode distance, and height. It was, however, associated with stronger selection acting on the number of branches. Given the fact that the substrate type was considered as a proxy of resources as nutrient or water and below ground space available in the ground, our results show that the more resources and space are limited (in bed rock environment), the stronger is the selection on number of leaves, internode distance, and height. This is not surprising because nutrient or water limitation is known to affect the biomass of plants (Poorter et al. 2012), through functional biomass allocation (Poorter and Nagel 2000). By affecting the topology of the fitness function of traits, the substrate type could thereby impact the reproductive benefit of divergent biomass allocation strategies within this snapdragon plant population. The vegetation coverage and density in conspecifics solely affected selection acting on height. Specifically, the advantage of being tall was smaller for plants surrounded by conspecifics and other species. This result is quite unexpected because one could expect stronger selection for taller plants surrounded by a dense vegetation as a result of competition for light. However, it is important to note that the effect of vegetation coverage (−0.068 ± 0.024) and density in conspecifics (−0.080 ± 0.032) on the selection acting on height was small in comparison to the linear selection gradient for this trait (*β* = 0.727 ± 0.011). Altogether, although the environmental drivers alter the ecological context that promotes selection within the population, they could be seen as hints to predict selection in populations experiencing different ecological conditions. Indeed, our analyses detected a positive selection acting on number of leaves only in the bed rock substrate type. This means that different populations with different proportions of substrate types might exhibit divergent selection regimes acting on number of leaves. Likewise, this population might exhibit a temporal fluctuation in selection pressures acting on height if the vegetation coverage and the density in neighbors fluctuate across years. Beyond the problem of causality, investigating fine scale spatial variation in selection within a population driven by ecological conditions represents an interesting starting point to predict the divergence in selection experienced by two populations at different locations, or the temporal fluctuation in selection in response to changing environment.

Such extrapolation of potential causality must nevertheless be taken cautiously. Direct causality between our environmental variables and selection cannot be assessed in the absence of mechanistic experimental approaches. Indeed, environmental variables may affect both the traits and fitness, thereby inducing a fitness‐trait covariance that could be mistaken for selection. Several studies pointed out this inherent problem to any investigation of natural selection in natura (Rausher 1992; Stinchcombe et al. 2002; Morrissey et al. 2012). However, the smooth‐tensor product between environmental variable and traits cannot be the product of an environmental‐induced trait‐fitness covariance in our approach because we also included the direct effect of the environment on the fitness function. As a result, the fitness function reflects the phenotype effect on fitness regardless of the impact of the environment on fitness. For example, fitness was significantly higher on the bed rock substrate than on the soil substrate for plants with a similar number of leaves, regardless of the direct negative or positive average effect of the substrate type on fitness. Moreover, we used a spatially explicit fitness function that controlled for any spatially varying unmeasured covariable potentially affecting the fitness‐traits‐environment relationship. Indeed, previous work shows that taking into account spatial autocorrelation in fitness in any model of selection controls for any *spatial* covariation between fitness and trait that would be the result of an unmeasured spatially variable ecological driver (Marrot et al. 2015).

The use of a spline‐based fitness function including a smooth‐tensor interaction between the environment and the traits revealed the spatial variation of selection associated with ecological agents in this wild population of snapdragon plants. Our findings demonstrate that microenvironmental variation at the scale of a meter can affect not only the fitness of individuals, but also the advantage of having a specific phenotype in an herbaceous plant. Our finding raises the hypothesis that different phenotypes might be selected at different locations at an extremely small spatial scale in wild plant populations, which could potentially maintain phenotypic variation in the presence of homogenizing gene flow. More studies linking natural selection and its ecological drivers in wild population are needed to develop a more mechanistic understanding of the ecological context shaping selection and ultimately evaluate how selection might change in response to environmental perturbations.

## AUTHOR CONTRIBUTIONS

BP designed the research program. BP, ML, JP, and PM contributed to the field sampling and data collection. PM analyzed the data. BP and PM wrote the manuscript.

## CONFLICT OF INTEREST

The authors declare no conflict of interest.

## DATA ARCHIVING

Data and R protocols used for the statistical analyses are available from the ZENODO Digital Repository https://doi.org/10.5281/zenodo.4698402.

Associate Editor: Dr. Adam Siepielski

Handling Editor: Dr. Andrew McAdam

## Supporting information

Supplementary material

Supplementary material

Supplementary material

Supplementary material
