# Supplementary material for: Natural selection fluctuates at an extremely fine spatial scale inside a wild population of snapdragon plants
Source: Evolution. 2021 Oct 1;76(3):658–66. doi: 10.1111/evo.14359 (PMC9291555; doi:10.1111/evo.14359)
Supplement: Supplementary file 1 — Supplementary material [file EVO-76-658-s002.docx]

**Supplementary Information 1: Evaluation of the substrate type and vegetation coverage in the field**


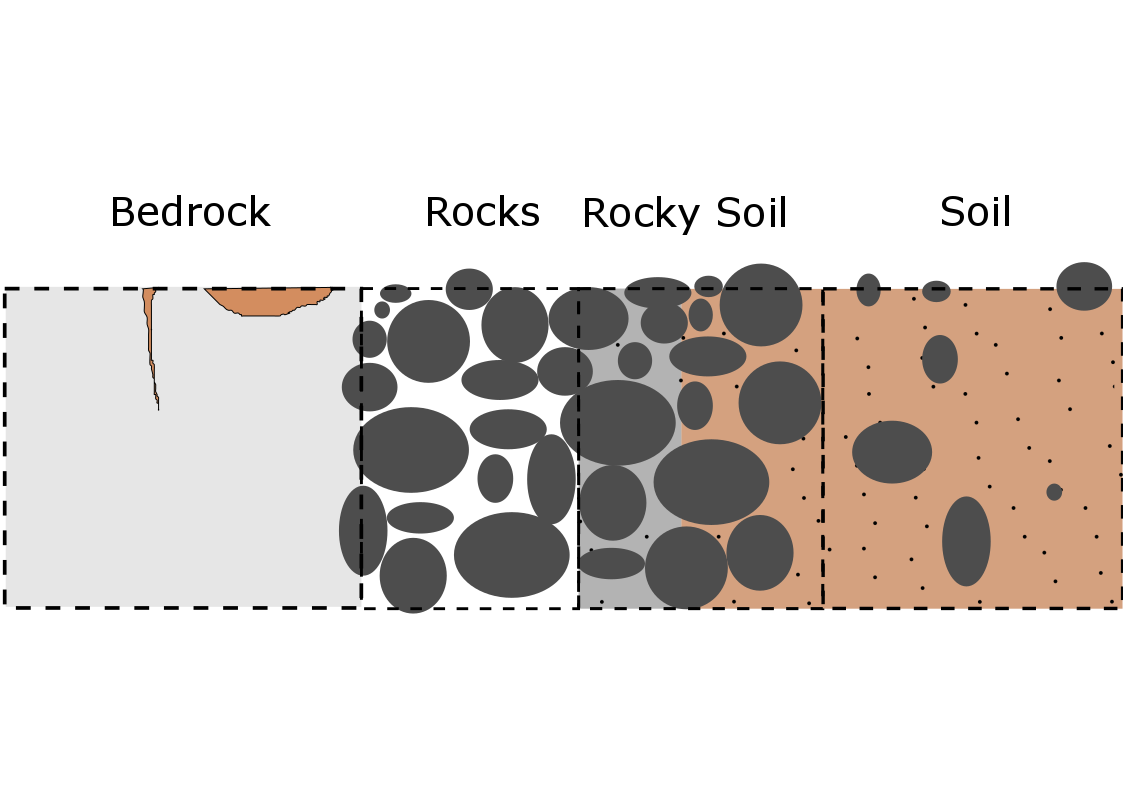


**Bedrock Large Rocks Rocky Soil Soil**

**Fig SI.1.1**. Estimation of the substrate type the plant is rooted in. Four substrate types are discriminated: (1) **Bedrock** (a large rock or rocky platform) where plants are defined by having limited root space. This includes plants growing in soil scrapes on the surface of a bedrock, in holes, and in cracks between bedrock slabs. (2) **Large** **Rocks** (rocks (pebbles) with no soil or small stones between them) where plants can be growing between large rocks, but no finer substrate can be observed between them. (3) **Rocky Soil** (rocks with visible soil or very fine pebbles that are becoming soil). The point is that the larger pebbles are sitting on top of / in a fine substrate. (4) **Soil** (soil, with no or few pebbles).


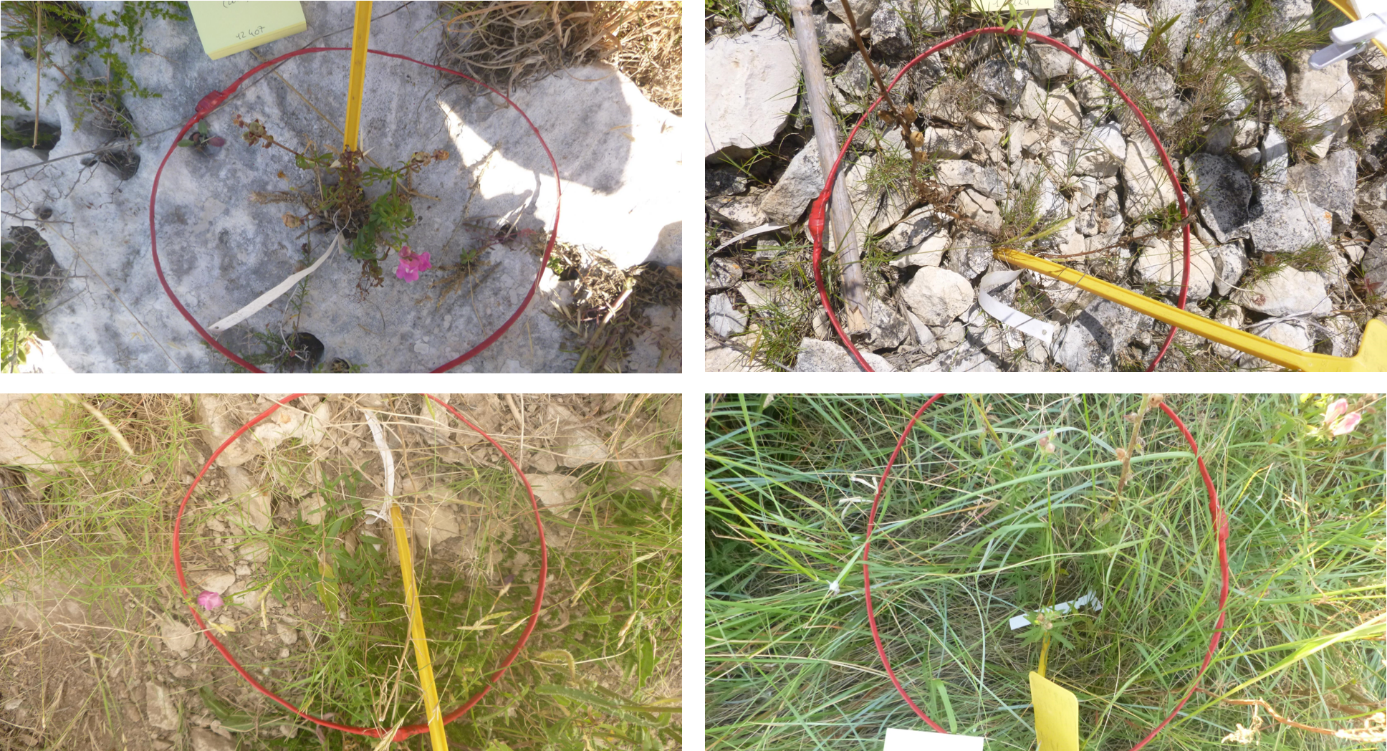


**D**

**C**

**B**

**A**

**Fig SI.1.2**: Photos of each substrate type observed on the field: **A** bedrock, **B** large rock, **C** rocky soil and **D** soil.


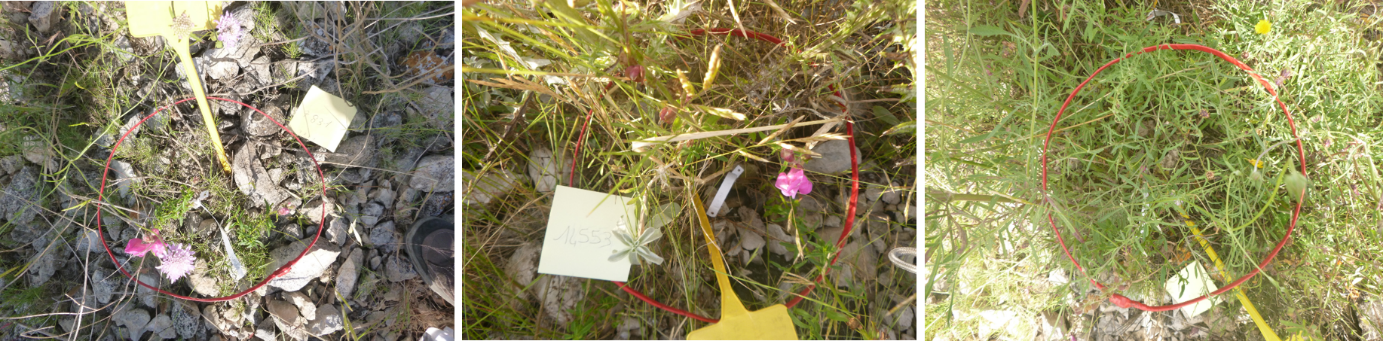


**C**

**B**

**A**

**Fig SI.1.3**: Photos of three different vegetation coverages observed on the field: **A** 10% vegetation coverage, **B** 50% vegetation coverage and **C** 90% vegetation coverage.
