# Supplementary material for: Natural selection fluctuates at an extremely fine spatial scale inside a wild population of snapdragon plants
Source: Evolution. 2021 Oct 1;76(3):658–66. doi: 10.1111/evo.14359 (PMC9291555; doi:10.1111/evo.14359)

**Supplementary Information 2: Statistical distribution of fitness (number of fruits), traits (number of leaves, number of branches, number of stems, internodes distance, height) and environmental variables (substrate type, vegetation coverage, conspecifics density).**

Red dashed line is representing the mean trait value in our population (mean value is indicated on the graph). On-the-graph information: percentage of the population presenting a trait value of zero.


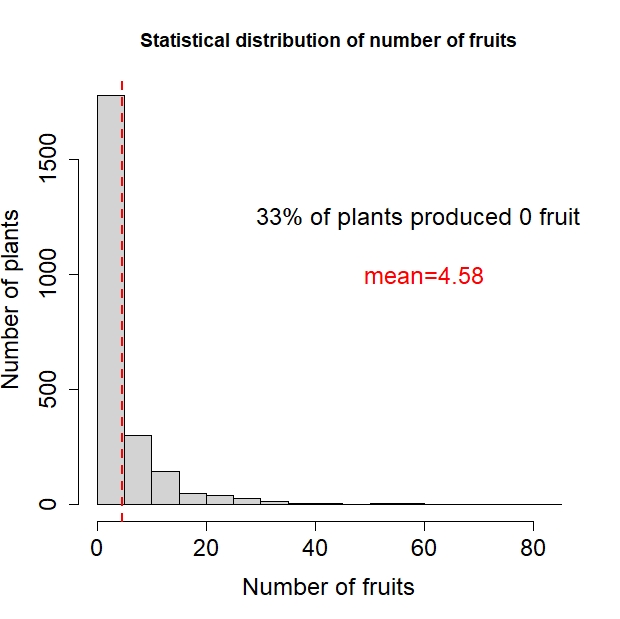


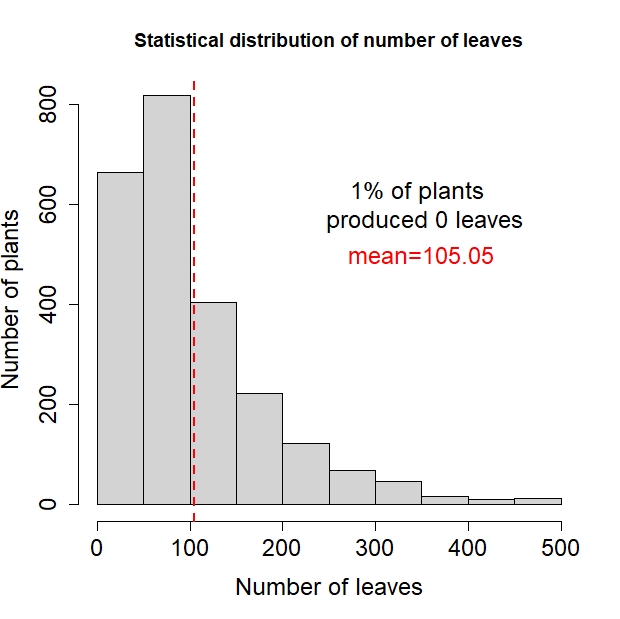


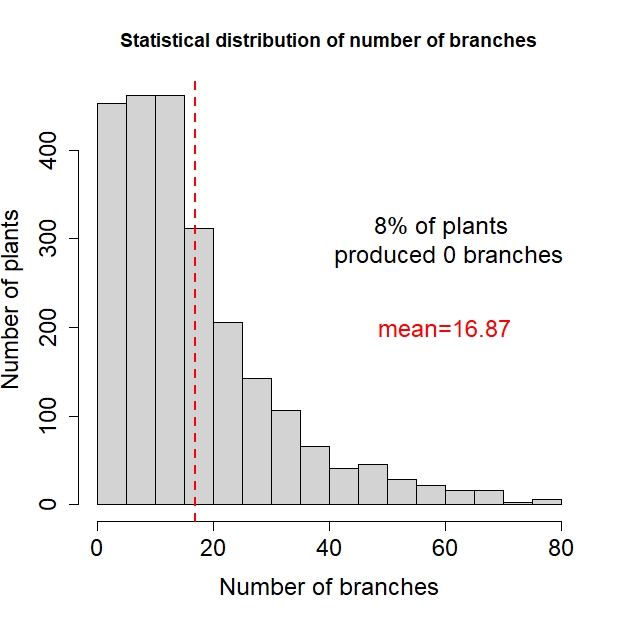


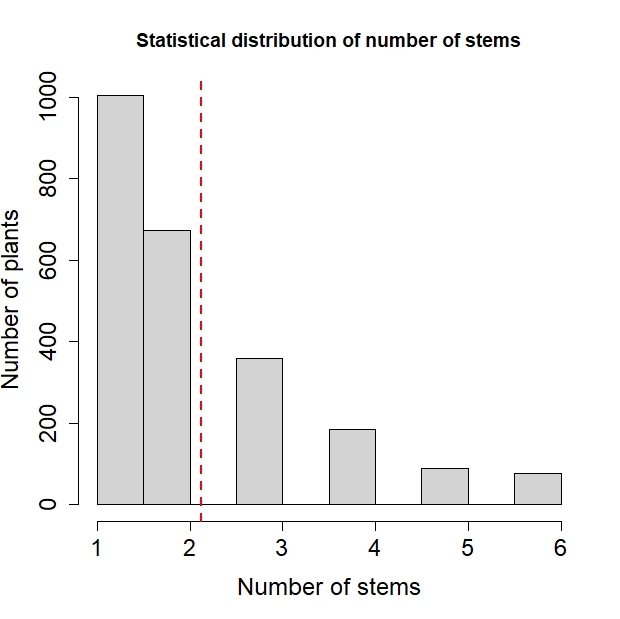


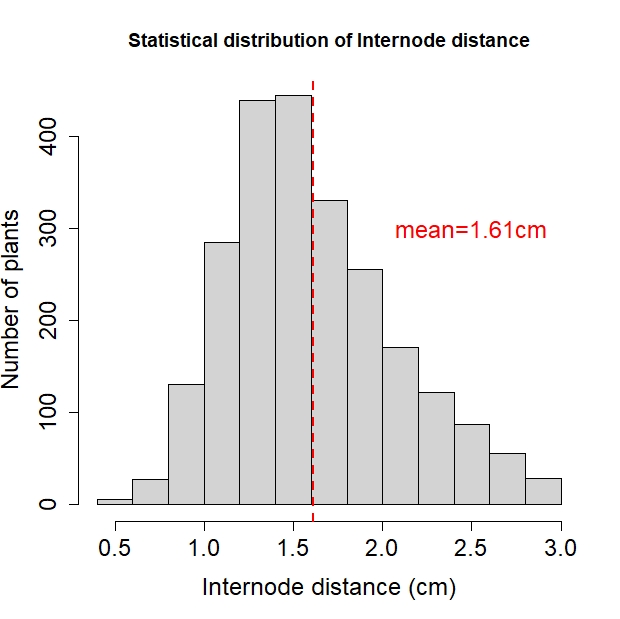


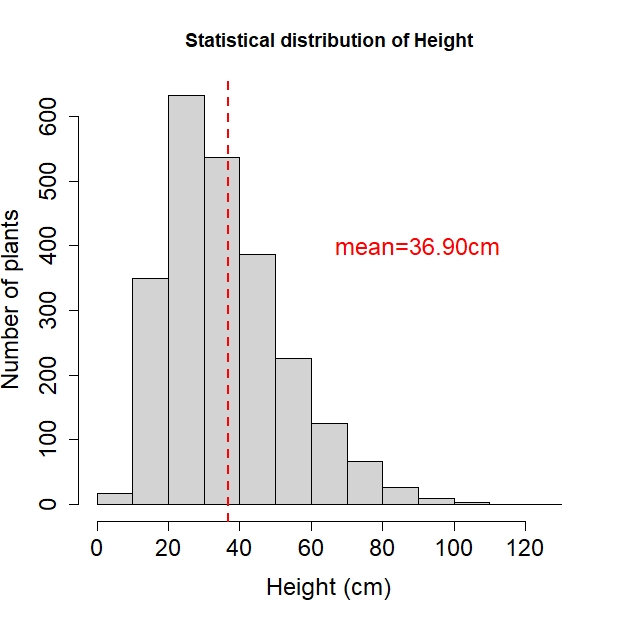


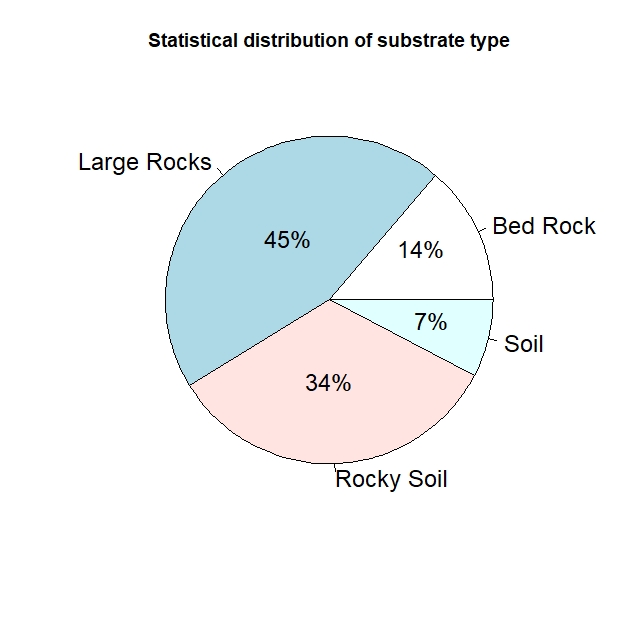


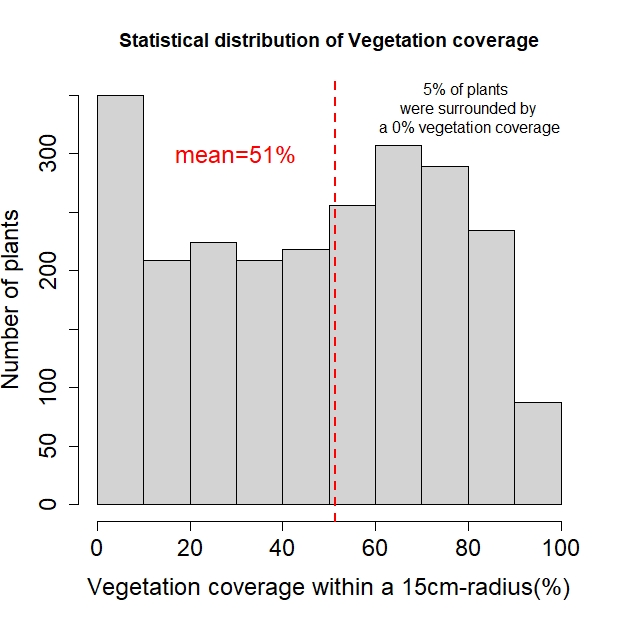


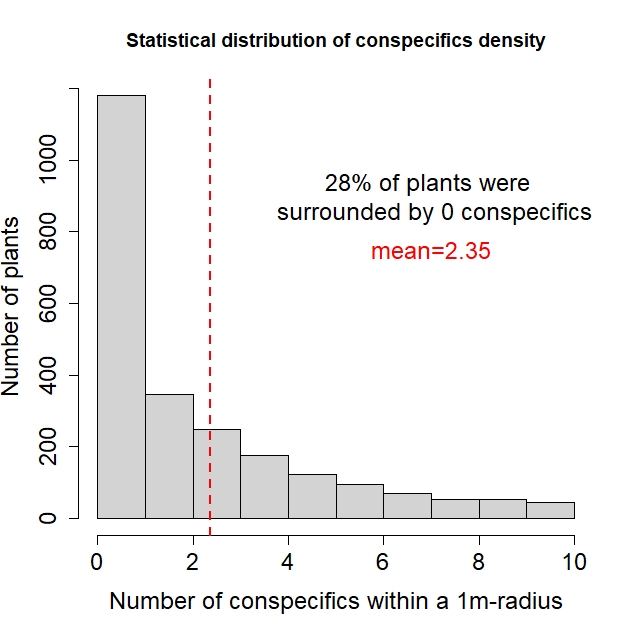

Supplement: Supplementary file 2 — Supplementary material [file EVO-76-658-s001.docx]
