# Supplementary material for: Natural selection fluctuates at an extremely fine spatial scale inside a wild population of snapdragon plants
Source: Evolution. 2021 Oct 1;76(3):658–66. doi: 10.1111/evo.14359 (PMC9291555; doi:10.1111/evo.14359)
Supplement: Supplementary file 3 — Supplementary material [file EVO-76-658-s004.docx]

**Supplementary information 3: Controlling for spatial autocorrelation**

The three environmental variables (substrate type, vegetation coverage and conspecifics density), the fitness (estimated by the number of fruits produced), and the five traits (number of leaves, number of branches, number of stems, internode distances and height) were characterized by an heterogeneous spatial distribution (Fig. SI2.1 and Fig. SI2.2).

Fitness was positively spatially autocorrelated (Moran test *P* < 0.001). In order to control for this effect in the fitness function (see Material and Method), we included a smooth tensor interaction term between latitude and longitude (Dormann et al., 2007) in our fitness function. As a result, we took into account any non-linear spatial dependency of fitness in the population. We tested that our latitude-longitude smooth tensor successfully controlled for spatial autocorrelation in fitness by estimating spatial autocorrelation in residuals from a model linking fitness to the latitude-longitude smooth tensor. We were confident to include the latitude-longitude smooth tensor in our fitness function in order to remove any spatial dependency in the fitness-traits-environment relationship because residuals were no longer spatially autocorrelated (Moran test *P* = 0.9915).


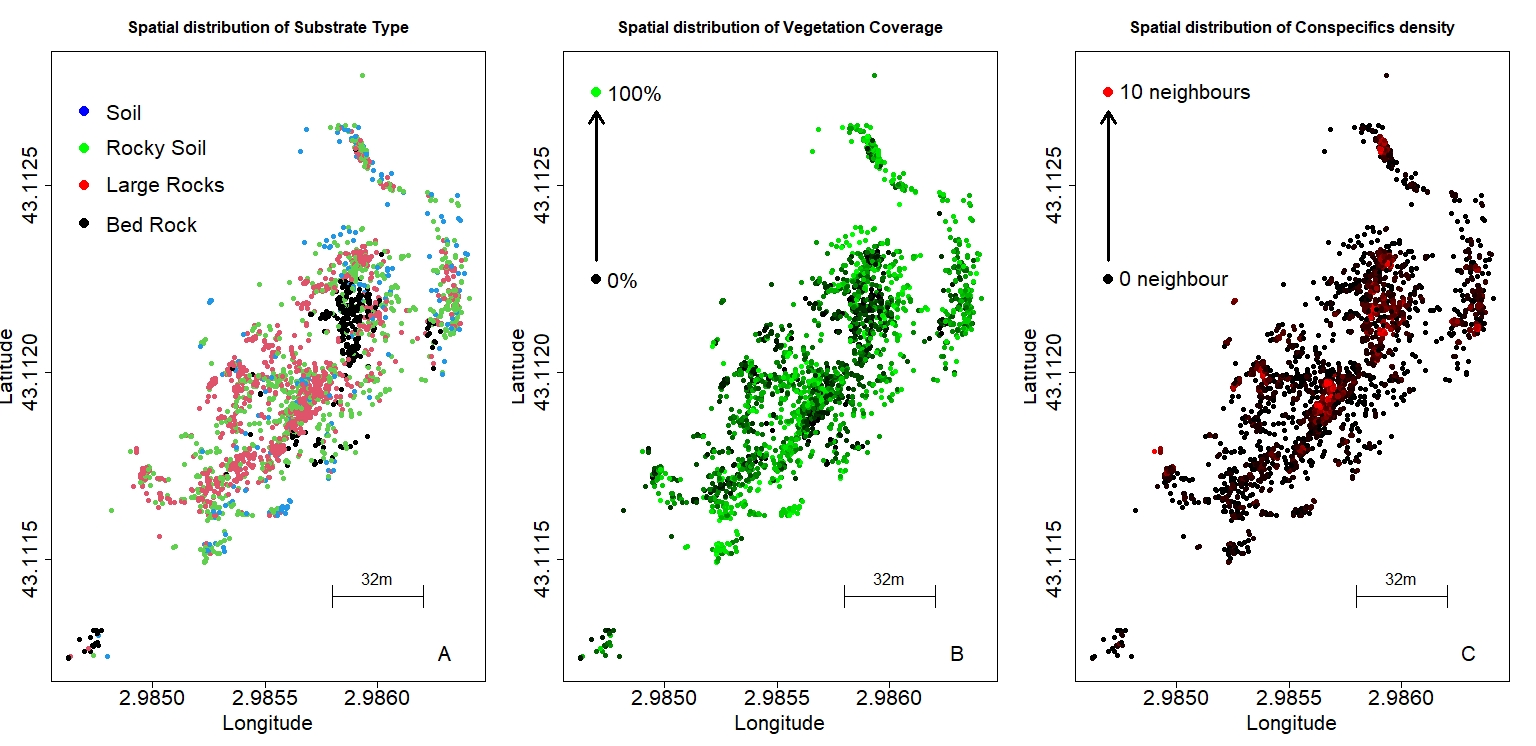


Figure SI.3.1: Spatial distribution Substrate type (A), Vegetation coverage (B) and Conspecifics density (C).

Spatial distribution of number of leaves

Spatial distribution of Fitness (number of fruits)


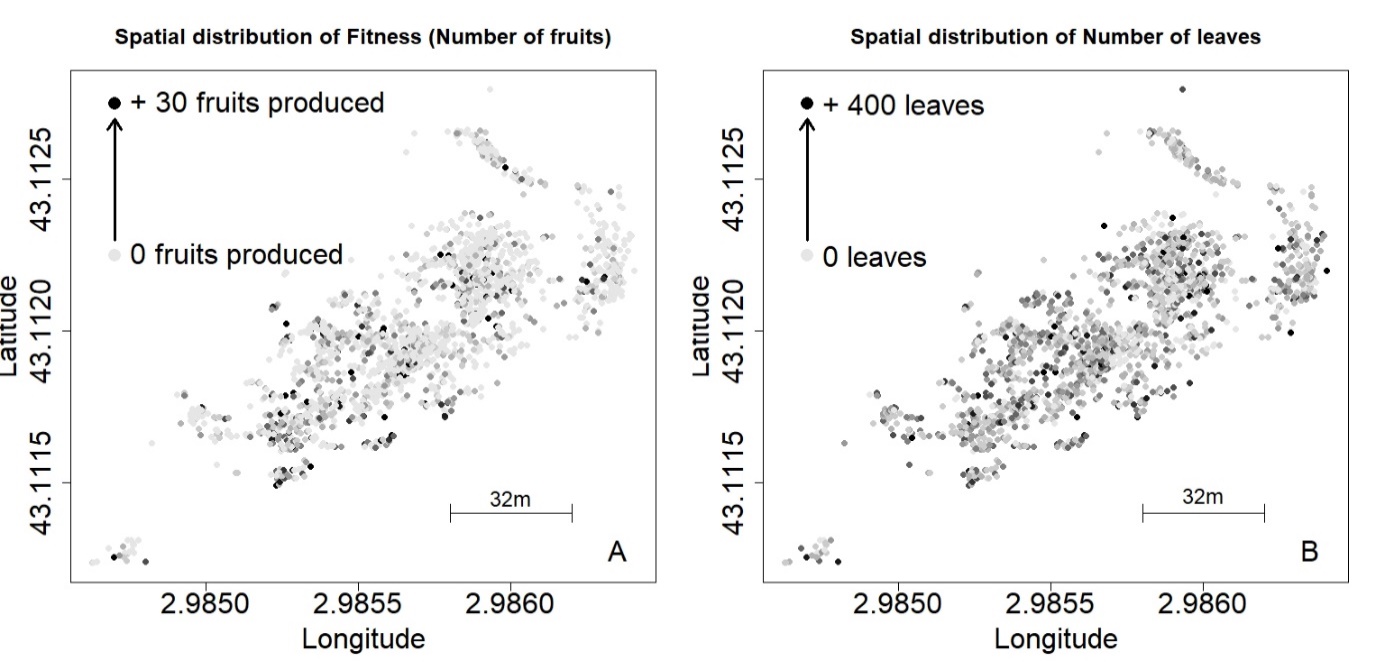

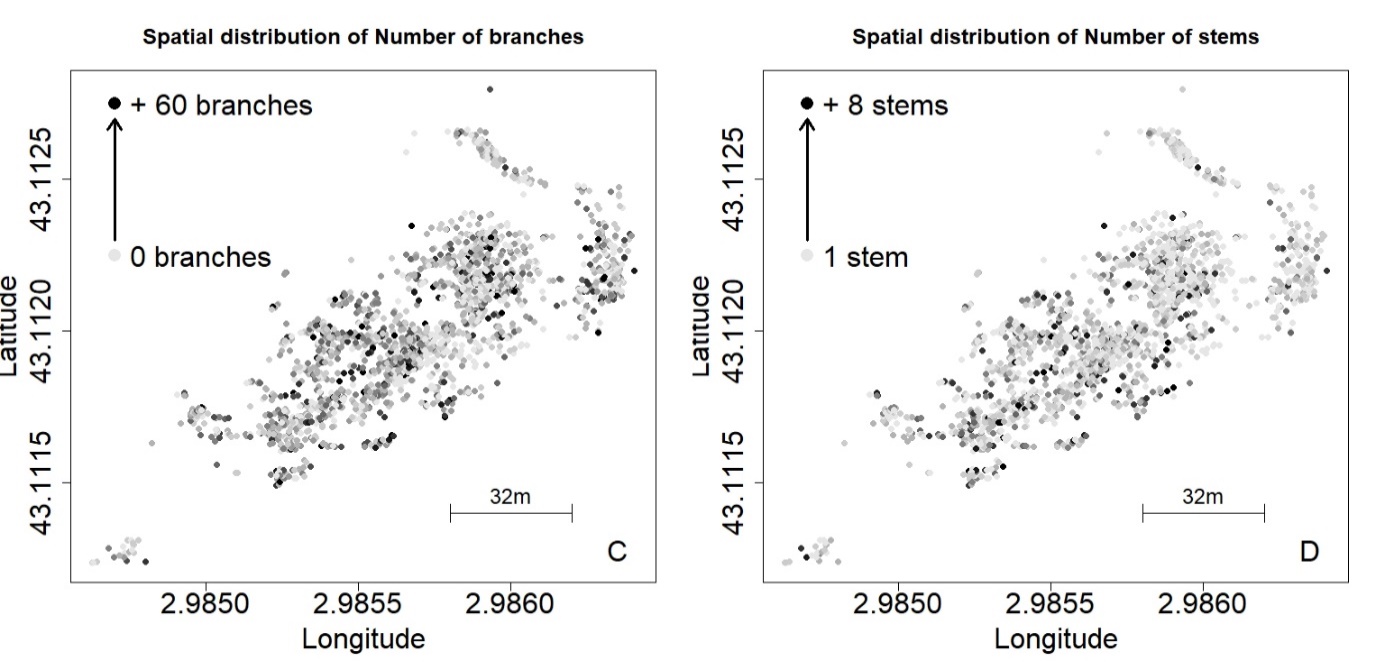

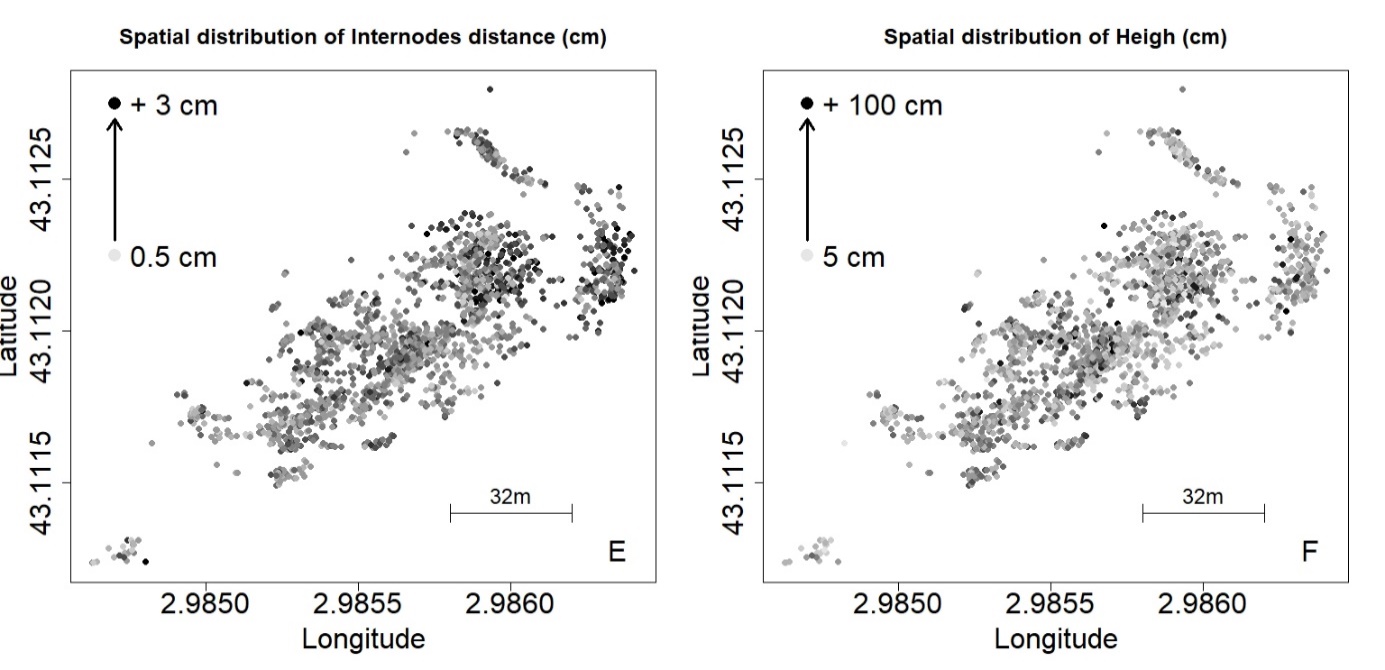


Spatial distribution of number of stems

Spatial distribution of number of branches

Spatial distribution of number of height

Spatial distribution of internode distance

Figure SI.3.2: Spatial distribution of fitness (A), number of leaves (B), number of branches (C), number of stems (D), internode distance (E) and height (F).
