# Supplementary material for: Natural selection fluctuates at an extremely fine spatial scale inside a wild population of snapdragon plants
Source: Evolution. 2021 Oct 1;76(3):658–66. doi: 10.1111/evo.14359 (PMC9291555; doi:10.1111/evo.14359)
Supplement: Supplementary file 4 — Supplementary material [file EVO-76-658-s003.docx]

**Supplementary Information 4:** **Fitted value of the fitness function predicted by the number of leaves, the number of branches, the number of stems, the internode distance and height.**

Note that these predicted values are extracted holding the non-focal variables constant from a generalized additive model (GAM) using the *ggeffects* (Lüdecke 2018) package in R.

**
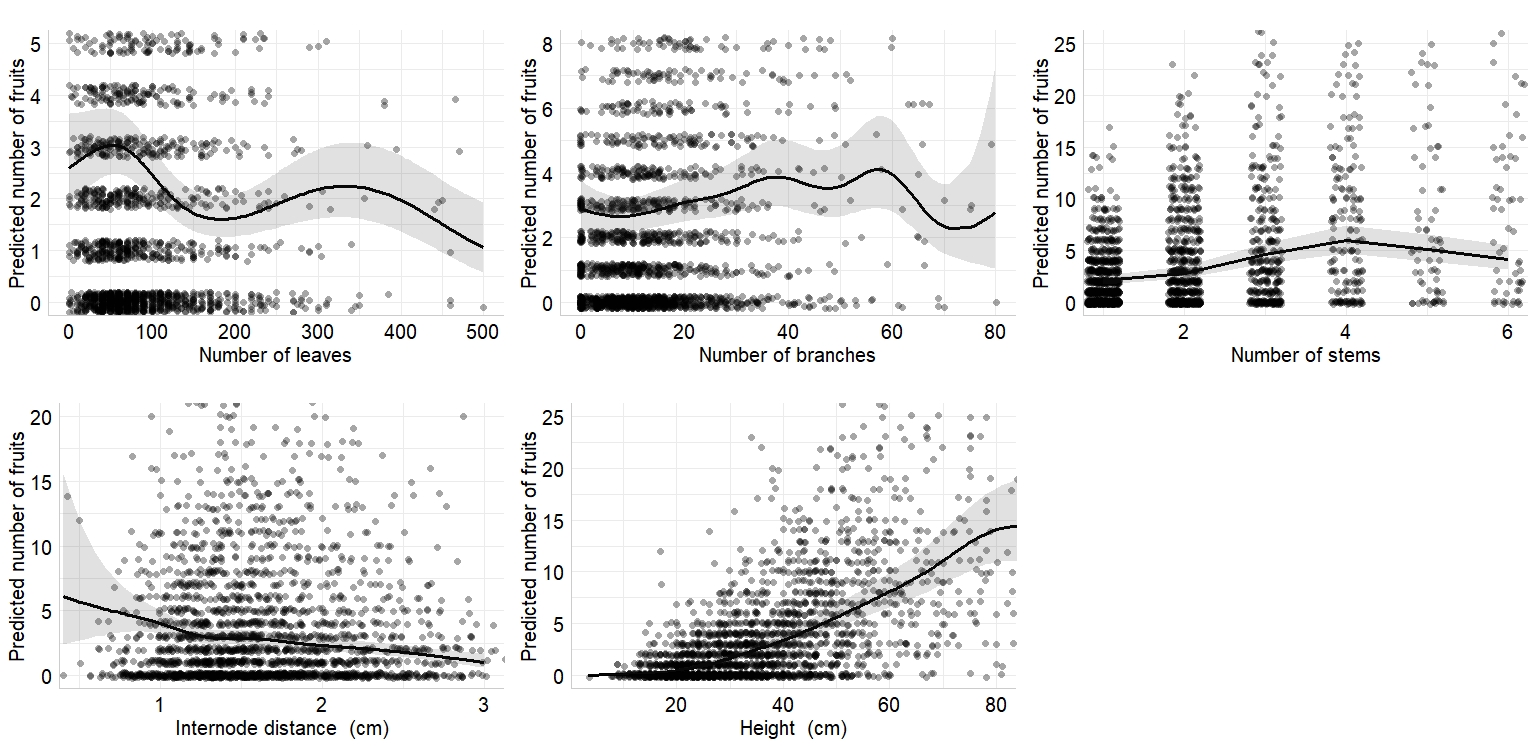
**

**References:**

Lüdecke D (2018). ggeffects: Tidy Data Frames of Marginal Effects from Regression Models. Journal of Open Source Software, 3(26), 772. doi: [10.21105/joss.00772](https://doi.org/10.21105/joss.00772)
